# Standardizing moderate- and vigorous-intensity exercise doses by physiological strain: an exploratory randomized cross-over study

**DOI:** 10.1007/s00421-026-06157-1

**Published:** 2026-03-09

**Authors:** Olli-Pekka Nuuttila, Piia Kaikkonen, Timi Malinen, Harri Sievänen, Tommi Vasankari, Heikki Kyröläinen

**Affiliations:** 1 The UKK Institute for Health Promotion Research, Tampere, Finland; 2 Faculty of Sport and Health Sciences, University of Jyväskylä, Jyväskylä, Finland; 3 Tampere Research Center of Sports Medicine, UKK Institute, Tampere, Finland; 4 Faculty of Medicine and Health Technology, Tampere University, Tampere, Finland

**Keywords:** Exercise prescription, Lactate threshold, Physiological strain, Training load

## Abstract

**Background:**

Current approaches to prescribing exercise intensity might not standardize physiological strain across individuals, potentially affecting training outcomes. Whether exercise guidelines applying a 1:2 duration ratio between moderate (MOD) and vigorous (VIG) activities equalize physiological strain is unclear. This exploratory study compared physiological strain and its variance across two intensities and three prescription methods.

**Methods:**

Thirteen habitually active males performed an incremental treadmill test as well as 40-min MOD and 20-min VIG sessions prescribed by: (1) absolute metabolic equivalents (ABS), (2) relative maximal oxygen uptake (VO_2max_) (REL), and (3) lactate thresholds (LT). Physiological strain was estimated using excess post-exercise oxygen consumption, individualized training impulse, post-exercise heart rate variability (HRV), blood lactate, and session rating of perceived exertion (sRPE).

**Results:**

Except for sRPE, estimated physiological strain was greater during VIG than MOD (*p* < 0.05) across all prescription methods. A general linear mixed model estimated that equivalent strain for most outcomes would have required MOD > 55% and VIG < 72% of VO_2max_. According to modified Levene’s test, variance in physiological strain did not differ significantly between prescription methods in MOD. In blood lactate, the variance was higher in VIG_REL_ than VIG_LT_ (*p* = 0.025), and HRV variance was greater in VIG_ABS_ than VIG_LT_ (*p* = 0.041).

**Conclusion:**

The 1:2 duration ratio underestimated physiological strain of VIG sessions regardless of prescription method. Based on the observed differences in variance, the appropriate prescription method may become increasingly important at higher intensities. However, analyses indicated substantial uncertainty in the magnitude of variance, highlighting the need for large-scale studies on the topic.

**Supplementary Information:**

The online version contains supplementary material available at 10.1007/s00421-026-06157-1.

## Introduction

Exercise dose is the aggregate outcome of frequency, intensity, and volume of activities (Wasfy and Baggish 2016). Within a single activity, the volume is typically quantified by time or distance covered (Mujika 2017). The intensity, which refers to the internal demand of an activity, can be classified with absolute (e.g., fixed metabolic equivalents (MET) (Iannetta et al. 2021), relative (e.g., %/maximum oxygen uptake (VO_2max_) (Jamnick et al. 2020), and threshold-based (e.g., lactate thresholds (LT) methods (Jamnick et al. 2020). The method applied for intensity determination depends often on the context and whether the focus is more on assessment of physical activity, health promotion or exercise training aimed at improving performance (Coates et al. 2023). In the population level guidelines of physical activity, target volumes are presented in time, while intensity is divided into moderate and vigorous levels (Piercy et al. 2018; Bull et al. 2020). The total physical activity dose is determined by the interaction between intensity and volume, where one minute of vigorous-intensity is considered equivalent to two minutes of moderate-intensity (Piercy et al. 2018; Bull et al. 2020), regardless of the frequency of activities.

General intensity standardization methods have evoked a lot of criticism during recent years (Iannetta et al. 2020, 2021; Jamnick et al. 2020; Meyler et al. 2021). The criticism mostly concerns the high heterogeneity of physiological responses, if intensity is fixed relative to maximum values of heart rate (HR) or VO_2_ (Iannetta et al. 2020; Jamnick et al. 2020; Meyler et al. 2021). Methods based on metabolic thresholds (ventilatory and/or lactate) and critical speed/power have been proposed as the primary options for intensity standardization (Jamnick et al. 2020; Meyler et al. 2021), which was also acknowledged in the recent consensus statement of American College of Sports Medicine (ACSM) and Exercise and Sport Science Australia (ESSA) (Bishop et al. 2025). Metabolic thresholds can be used to divide intensity domains into moderate, heavy, and severe, all of which have distinct physiological characteristics (Jamnick et al. 2020). The moderate-intensity domain induces low homeostatic disturbance as well as stable VO_2_ and blood lactate kinetics, while steady state is delayed in the heavy intensity domain and not attained in the severe intensity domain (Jamnick et al. 2020). Since it is known that thresholds in relation to maximum are highly individual (Iannetta et al. 2020; Nuuttila et al. 2025), it is questionable whether general fixed methods (absolute or relative) can standardize exercise dose between individuals. This lack of reliability may have significant implications when evaluating acute and chronic dose-response associations of physical activity or exercise.

To quantify and equalize the dose of different exercise intensities and durations, the concept of training load has been developed (Passfield et al. 2022; Impellizzeri et al. 2023). While the training load can be further divided into external and internal load, particularly the internal load is considered to reflect the physiological strain of a given exercise affecting acute responses and chronic training adaptations (Impellizzeri et al. 2023). Examples of widely adopted training load metrics include those that combine internal load (e.g., exercise HR) with duration, such as training impulse (TRIMP), and those that combine perceived exertion with exercise duration, such as the session rating of perceived exertion (sRPE) (Borresen and Ian Lambert 2009). In addition to these metrics, physiological responses during the recovery phase of an exercise bout have also been proposed to reflect the internal load of the preceding activity. These responses, relating to the acute disturbance of homeostasis after exercise, include excess post-exercise oxygen consumption (EPOC) (Mann et al. 2014b) and heart rate variability (HRV), the non-invasive marker of autonomic nervous system activity (Kaikkonen et al. 2010). Although traditional training load metrics have their limitations and their validity has also been questioned (Passfield et al. 2022), the exercise doses defined by them have been associated with training adaptations in many studies (Manzi et al. 2009; Wallace et al. 2014a; Sanders et al. 2017) supporting their relevance in fitness-related dose-response analyses. Several studies have reported how blood lactate responses are affected by the exercise intensity prescription method (Lansley et al. 2011; Meyler et al. 2023; Pacitti et al. 2025). To the best of our knowledge, none have examined how well these methods can standardize the physiological strain as measured by other training-load-related metrics. In addition, to our knowledge, previous studies have not investigated how well a 1:2 duration ratio, commonly used to standardize doses between moderate and vigorous intensities (Piercy et al. 2018; Bull et al. 2020) or between moderate and heavy intensity domains (Lucia et al. 2003), would equalize the physiological strain of these intensities. Therefore, this exploratory study assessed physiological responses during and after moderate- and vigorous-intensity exercises performed with absolute, relative, and lactate threshold-based standardization in habitually active males. The main focus was to (1) assess how the 1:2 method equalizes physiological strain when three different prescription methods are used, (2) analyze whether the lactate threshold-based prescription could diminish variance in physiological strain. It was hypothesized that physiological strain would remain higher in VIG than MOD across prescription methods when using the 1:2 ratio, while the LT-based prescription would reduce between-participant variance compared with ABS and REL.

## Methods

### Participants

A total of 15 participants were recruited through the communication channels of the University of Jyväskylä. The required sample size was estimated based on previous studies that have reported statistically significant differences in variances of physiological metrics across various intensity prescription methods (Lansley et al. 2011; Meyler et al. 2023). Inclusion criteria required participants to be healthy males aged 20–50, accustomed to moderate-intensity physical activity, but not currently participating in regular aerobic training (3 × 30 min per week: American College of Sports Medicine et al. 2022). Exclusion criteria included diseases, medications, and symptoms (e.g., cardiovascular or musculoskeletal) that could be considered risk factors for participation or could potentially affect the outcomes measured in the study (e.g., HR-based variables). The target group was chosen to represent the general population rather than people at either end of the spectrum. Because the sample size was relatively small, only males were included to eliminate potential menstrual-cycle–related effects (i.e., additional variance) on the measured outcomes. The eligibility of the participants was confirmed with a custom-made health questionnaire and resting electrocardiography (ECG) that was approved by a physician. The descriptive characteristics of the participants included in the final analysis are presented in Table 1. One participant had an abnormal ECG and was referred for further examinations, and one participant could not participate in all required testing sessions due to scheduling issues. Thus, the final sample consists of thirteen participants. All participants gave their written informed consent to participate. The study protocol was approved by the Ethics Committee of the University of Jyväskylä (755/13.00.04.00/2024).

**Table 1:** Baseline descriptive characteristics (mean ± SD) of the participants

	*n* = 13
Age (yrs)	42.8 ± 6.8
Height (cm)	180.2 ± 6.4
Body mass (kg)	89.0 ± 14.9
Body mass index (kg/m^2^)	27.3 ± 3.8
Fat percentage (%)	18.3 ± 4.6
Resting HR (bpm)	60.3 ± 6.0
Maximum HR (bpm)	185.8 ± 9.9
vLT1 (km/h)	6.5 ± 0.7
vLT2 (km/h)	9.1 ± 1.6
vPeak (km/h)	12.6 ± 1.7
VO_2max_ (ml/kg/min)	41.4 ± 6.6
VO_2max_ classification (1–7)*	4.7 ± 1.4
Daily steps (number)	6788 ± 2038

HR = heart rate; vLT1 = speed at the first lactate threshold; vLT2 = speed at the second lactet threshold; vPeak = peak treadmill test speed; VO_2max_ = maximum oxygen uptake. *Based on age-specific reference values by Shvartz and Reibold (Shvartz and Reibold 1990)

### Study design

The six-week study period consisted of a preliminary assessment of cardiorespiratory fitness (visit 1) and six exercise sessions (visits 2–7) that were performed in a randomized order (cross-over setting). All exercise sessions were performed at the same time of the day (± 2 h) and primarily at the same time of the week in the same laboratory conditions. Participants were asked to refrain from strenuous activities on the day preceding laboratory visits but otherwise to continue with their regular physical activity patterns throughout the study period. Participants were also asked to arrive for tests/exercises in a similar nutritional state and to avoid heavy meals and caffeine for at least three hours beforehand. To avoid any significant adaptations due to training sessions and a potentially increased amount of physical activity, the sessions were scheduled once a week with only a few exceptions. In addition to testing, the participants’ physical activity was monitored with a tri-axial accelerometer (Movesense, Suunto, Vantaa, Finland) throughout the study period.

## Measurements

### Visit 1: incremental treadmill test and VO_2max_ verification test

*The incremental treadmill test* was performed on a motorized treadmill (Telineyhtymä Oy, Kotka, Finland). The test protocol consisted of 3-min stages and 1 km/h increments until volitional exhaustion. The current protocol was chosen to assess LTs and maximum performance within the same test (Bentley et al. 2007). It is also the recommended testing protocol according to the general Finnish fitness test guidelines (Keskinen et al. 2018). The treadmill was stopped between stages (~ 15–20 s) to collect fingertip blood samples into capillary tubes (20 µl) for blood lactate analysis. The starting speed of the test was fixed to 4 km/h to ensure reliable recognition of the first LT (LT1) for all participants. The test was performed by walking until 7 km/h, after which the participants were asked to run. The inclination of the treadmill was kept at 1% during the test. HR (Polar H10, Polar Oy, Kempele, Finland) and VO_2_ (Jaeger VyntusTM CPX, CareFusion Germany 234 GmbH, Hoechberg, Germany) were monitored continuously during the test. HR was analyzed as the last 30-s average and VO_2_ as the last 60-s average in each stage. In addition, the participants were asked to rate their perceived exertion (6–20) (Borg 1982) after each stage. Blood lactate concentrations were analyzed afterwards with a Biosen C-line lactate analyzer (EKF Diagnostic, Magdeburg, Germany). Before each analysis, the analyzer was calibrated (one-point calibration) with a standard lactate/glucose solution (12 mmol/l). Accuracy (CV%) of the blood lactate analyses is according to the manufacturer ≤ 1.5%.

VO_2max_ was defined as the highest 30-s average observed during the test. In turn, the peak treadmill test speed (vPeak) was defined as the highest completed stage speed, or if the stage was not finished, as the speed of the last completed stage (km/h) + (running time (s) of the unfinished stage – 30 s) / (180–30 s) · 1 km/h that takes into account the short break between stages. The HRmax was defined as the highest 5-beat average during the test according to default settings of Kubios HRV Scientific software version 4.0 (Kubios Oy, Kuopio, Finland).

Lactate thresholds were set according to criteria presented in Nuuttila et al. (2025): LT1 was set at 0.3 mmol/L above the lowest lactate value observed during the test and the second lactate threshold (LT2) at the intersection point between two linear models: (1) a linear model between LT1 and the following measured lactate point; (2) a linear model for the lactate points with the lactate increase of at least 0.8 mmol/L.

*A verification test* was performed to ensure the achievement of VO_2max_. The protocol for the verification test was adapted from the review article of Schaun (Schaun 2017). The verification test started 10 min after the end of the incremental test. The recovery period between the tests was passive. The verification test started with a 3-min warm-up (2 min 50%/vPeak, 1 min 70%/vPeak), after which the treadmill speed was set at 1 km/h above the vPeak. The test continued until volitional exhaustion. VO_2max_ and HRmax were defined according to the same criteria as in the incremental test. Blood lactate samples were also taken before and after the verification test. The greater VO_2_ value achieved either in the incremental test or in the verification test was regarded as the individual’s VO_2max_.

### Visits 2–7: exercise sessions

#### Session design

Exercise sessions consisted of three 40-min moderate-intensity exercises (MOD) and three 20-min vigorous-intensity exercises (VIG). The intensities for the sessions were individually defined according to three different prescription criteria so that each method was utilized once for moderate and vigorous exercises:

1. Absolute intensity (ABS) was identical for all participants. Walking/running speeds were defined according to a compendium of physical activities (Herrmann et al. 2024). MOD_ABS_-session (5.9 km/h) corresponded to 4.8 MET for walking, while VIG_ABS_-session (7.3 km/h) corresponded to 7.8 MET for running. These speeds were also considered surrogates of “brisk walking” and “jogging”.
2. Relative intensity (REL) was defined as relative to VO_2max_. MOD_REL_-session corresponded to 54% of VO_2max_, while VIG_REL_-session corresponded to 77% of VO_2max_. The exact walking/running speed was defined as the closest speed at the target VO_2_/VO_2max_.
3. LT-based intensity (LT) was defined relative to the speed at LT1 and LT2. MOD_LT_-session corresponded to 90% of the speed at LT1, while VIG_LT_-session corresponded to 50% of the difference between LT1 and LT2.

The rationale for ABS- and REL-intensities was to apply halfway intensity within a domain as defined by ACSM (American College of Sports Medicine et al. 2022). The halfway intensity was deemed appropriate, because it most accurately reflects the target intensity and mitigates both potential errors in intensity determination and the effects of daily fluctuations in physiological responses. For LT-based prescriptions, the halfway method was applied for the vigorous (heavy-intensity domain) session, whereas for the moderate-intensity domain, there is no clear lower boundary that would allow the midpoint to be determined. Therefore, 90% of the speed at the LT was considered to represent a typical exercise intensity within this domain, with the corresponding physiological responses assumed to be appropriate. Furthermore, similar prescriptions for REL and LT have been applied previously in a comparable study design (Meyler et al. 2023). The 1:2 ratio between moderate- and vigorous-intensity session durations was designed to align with the ratio used in the general physical activity guidelines of WHO (Bull et al. 2020) and ODPHP (Piercy et al. 2018). A similar ratio is also used in the training impulse model of Lucia et al. (2003)

#### Assessments within a session

**Fig. 1:**
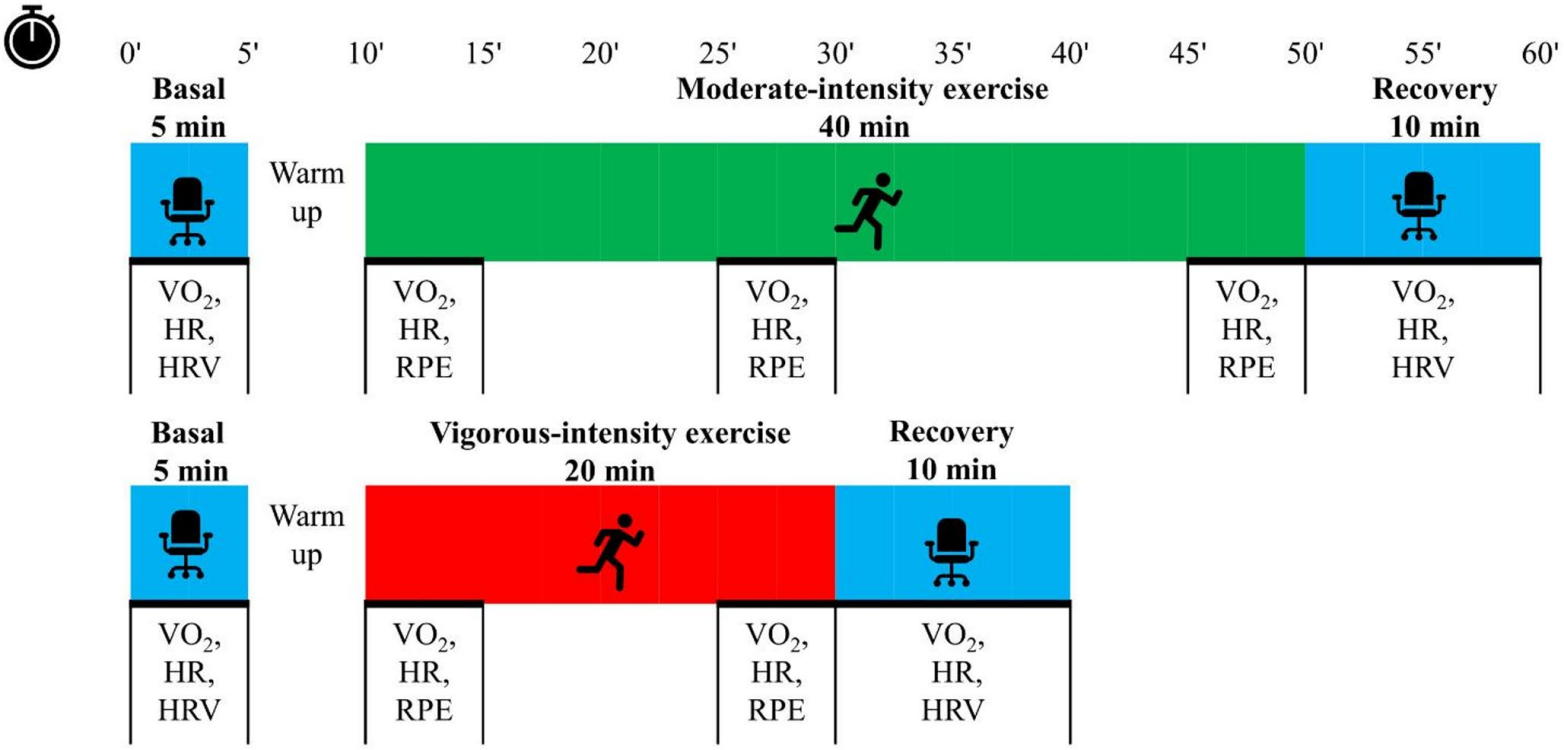
Structure of moderate- and vigorous-intensity exercises and pre- and post-exercise assessments

Demonstration of exercise session structure and analysis segments within a session is presented in Fig. 1.

##### Pre-exercise

Each session started with an assessment of body mass and a 5-min basal measurement of VO_2_ (Jaeger VyntusTM CPX, CareFusion Germany 234 GmbH, Hoechberg, Germany), HR, and HRV (Polar H10) in a seated position. The 2-min average of VO_2_, HR and HRV that started two minutes after the onset of the recording was regarded as the basal value to allow sufficient stabilization period for HR-related variables (Krejčí et al. 2018) and to minimize the impact of end-of-recording anticipation on the results. The resting assessment was followed by a 3-min warm-up that was performed on a treadmill 2 km/h below the individual LT1. Blood lactate was measured before and after the warm-up.

##### Exercise

One of the six exercises was performed after the warm-up. HR was recorded continuously, while VO_2_ was recorded during the first 5 min, from 15 to 20 min, and from 35 to 40 min (only MOD). VO_2_ was not recorded continuously due to comfort reasons. RPE was rated on a 6–20 scale at the end of each VO_2_ recording segment. The treadmill was stopped before the start of a 5-min VO_2_ recording to attach the mask. Blood lactate was measured immediately after the exercises. HR and VO_2_ were averaged over the last two minutes of each concurrent recording segment.

##### Post exercise

The exercises were followed by a 10-min recovery measurement of VO_2_, HR, and HRV. The participants sat immediately after the exercise on a chair that was brought on the treadmill. The breathing rate was not controlled during the recovery period, and participants were allowed to breathe spontaneously. The results were analyzed as 30-s averages across the recovery period.

All HR and HRV analyses were performed using Kubios HRV scientific software (version 4.0) with default settings and automatic data correction (Lipponen and Tarvainen 2019). The natural logarithm of the root mean square of successive differences (LnRMSSD) was used as the primary HRV parameter due to its validity as a marker of parasympathetic nervous system activity (Uusitalo et al. 1996; Hilz and Dütsch 2006). Furthermore, it is less sensitive to variations in breathing rate than the high‑frequency spectral component (Penttilä et al. 2001), making it a suitable HRV metric for this setting.

### Training-load-related metrics

Several different training load-related metrics were calculated based on the data collected during the exercise and recovery phase. The chosen parameters were considered to reflect the physiological strain of exercise from slightly differing perspectives.

- *Individualized TRIMP (iTRIMP)* was analyzed according to Manzi et al. (2009). While the original TRIMP method, as proposed by Banister (1991), considers ΔHR (HRexercise − HRrest /HRmax − HRrest) with a fixed multiplying factor, in the version of Manzi et al., the multiplying factor is individually defined based on exponential model for blood lactate vs. fractional HR elevation from an incremental treadmill test. In the current analysis, the TRIMP was analyzed as a sum of second-by-second values.
- *sRPE* (Foster et al. 1996) was formed by multiplying the estimated RPE value with the session duration. Participants rated each session on a 0–10 Foster’s sRPE scale after the 10-min recovery phase.
- *Post-exercise HRV* was derived as an average of the two first minutes of recovery, in line with Kaikkonen et al. (2010). LnRMSSD was considered the primary HRV parameter.
- *EPOC magnitude* was calculated as absolute VO_2_ (L) like Mann et al. (2014). EPOC was analyzed from the consecutive 30-s averages of the 10-min recovery phase. Basal values were individually pooled from all six sessions to ensure a more reliable reference.
- *Post-exercise blood lactate* was also regarded as a marker of physiological strain similar to Scharhag-Rosenberger et al. (2010)

The reliability of these outcomes (coefficient of variation (CV) has been reported in previous studies assessing responses to repeated exercise sessions: sRPE = 28.1% (Wallace et al. 2014b), post-exercise HRV 15.9% (Al Haddad et al. 2011), EPOC magnitude 8.0% (Mann et al. 2014a), blood lactate 15.6–23.1% (Pacitti et al. 2025). No suitable references were identified for the iTRIMP, but the CV values reported for Banister’s TRIMP (15.6%) (Wallace et al.2014b) were considered comparable to those expected for the iTRIMP.

### Physical activity monitoring

Physical activity was recorded throughout the study period with a tri-axial accelerometer (Movesense) and an accompanying mobile application (Exsed 2, UKK Terveyspalvelut Oy, Tampere, Finland). The participants were asked to wear the accelerometer on their right hip during the waking hours. The accelerometer data was analyzed in 6-s epochs using a validated mean amplitude deviation method (Vähä-Ypyä et al. 2015). The physical activity-related results provided by the application and used in the current analysis included the daily volume of moderate-to-vigorous-intensity (MVPA) activities (≥ 3.0 MET) and the number of steps taken. Days containing at least 10 h of wear time were considered valid in line with Norha et al. (2023). The results were analyzed as an average of the whole study period for the description of general activity level, but they were also analyzed week-by-week to assess possible changes in activity patterns during the study period.

### Nocturnal heart rate

Nocturnal HR was recorded with Firstbeat Bodyguard 2 (Firstbeat Technologies Oy, Jyväskylä, Finland) between the visits 1 and 2. The HR was analyzed as the average of the whole sleep period (as recorded in the diary). Nocturnal recordings were considered to be reliable and also comparable to resting assessments immediately after waking up (Nuuttila et al. 2024). The data was analyzed using Kubios HRV scientific software with default settings and an automatic data correction algorithm (Lipponen and Tarvainen 2019). The average resting HR of all recorded nights was used in the calculation of HR reserve.

### Statistical analysis

The normality of the data was analyzed using the Shapiro-Wilk test, and sphericity was examined using Mauchly’s test. To assess the effects of exercise prescription method (ABS, REL, LT) on responses to different exercise sessions, a two-way repeated measures ANOVA was applied with the prescription method and time as within-subject factors. These analyses were performed separately for MOD and VIG sessions. VO_2_, HR, and HRR analyses were performed with absolute values, but they are presented with relative values to allow comparison for ACSM intensity domains (American College of Sports Medicine et al. 2022). Training-load-related metrics were compared with intensity (MOD, VIG) and prescription method as within-subject factors, while session characteristics were compared between all six sessions. The Bonferroni post hoc test was used for pairwise comparisons of these analyses, and potential interactions were examined with simple contrasts between the first and the last time points. The variables that were not normally distributed were analyzed using the Friedman test (multiple comparisons) or the Wilcoxon signed rank test (paired analyses). The Greenhouse-Geisser correction was applied to outcomes that did not meet the sphericity assumption.

Inter-individual variance within training-load-related parameters was analyzed with the modified Levene’s test by performing pairwise comparisons for each session (Brown and Forsythe 1974). The equivalence of variances was examined using a two one-sided tests (TOST) procedure with pre-specified equivalence margins of ± 30% for the variance ratio. 30% was used as a cut-off, since it was comparable to the highest CV (sRPE) reported for the current main outcomes. A 90% confidence interval for the variance ratio was obtained using paired bootstrapping (5,000 resamples) to account for within-subject dependence. To ensure accuracy despite the skewed distribution of variance ratios, confidence intervals were derived using the bias-corrected and accelerated (BCa) method.

Furthermore, a generalized linear mixed-model analysis was performed to assess the effects of exercise intensity (ABS, REL, LT) and duration (20–40 min) on training-load-related metrics and to compare estimated physiological strain across the whole moderate- and vigorous-intensity spectrum. Model estimates were based on six intensity-duration pairs from each participant accounting for 78 pairs in total. For these analyses, mean VO₂ from all sessions was normalized according to METs, %VO_2_max, or LTs. Each training-load–related metric was then used as the dependent variable, assuming a Gamma distribution with a log link. Normalized exercise intensities and durations were included as fixed factors, and participants were entered as random effects to account for repeated measures. Model performance was evaluated using both the marginal and conditional pseudo-R². The marginal R² reflects the proportion of variance explained by the fixed effects alone, while the conditional R² reflects the proportion of variance explained by both fixed and random effects.

Effect sizes (ES) were derived to estimate the magnitude of effects in main outcomes and comparisons. Partial η^2^ (0.01 = small, 0.06 = medium, 0.14 = large) was analyzed for main effects and interactions, while Cohen’s d (0.20 = small, 0.50 = medium, 0.80 = large) was analyzed for pairwise comparisons. Statistical analyses were performed using IBM SPSS Statistics v.30 program (SPSS Inc, Chicago, IL, USA) and Microsoft Excel 2016 (Microsoft Corporation, Redmond, WA, USA).

## Results

There were no differences (*p* > 0.05) in the mean of daily steps or volume of MVPA between the first week of the study period (7201 ± 2065 steps, 77 ± 24 MVPA min) and any of the following weeks (ranges: 5847–7444 steps, 64–79 MVPA min). Regarding the effects of order in the basal assessments, VO_2_ during the warm-up was the only parameter that changed significantly (decreased, *p* < 0.05) from the first session.

### Session characteristics

Descriptive characteristics of different sessions are presented in Table 2. All vigorous-intensity sessions were performed at greater speed than moderate-intensity sessions (*p* < 0.05), except for VIG_ABS_ which was not different compared to MOD_REL_. MOD_REL_ was performed at greater speed (*p* < 0.05) than MOD_ABS_ and MOD_LT_, while VIG_REL_ was performed at greater speed (*p* < 0.05) than VIG_ABS_ and VIG_LT_. The speed did not differ between MOD_ABS_ and MOD_LT_ or VIG_ABS_ and VIG_LT_. Blood lactate concentration did not change from pre to post after MOD_ABS_ (-0.08 ± 0.41 mmol/L) or MOD_REL_ (0.20 ± 0.53 mmol/L), while it decreased (*p* = 0.02) after MOD_LT_ (-0.15 ± 0.19 mmol/L). In turn, blood lactate concentration increased (*p* < 0.05) from pre to post after VIG_ABS_ (1.33 ± 1.76 mmol/L), VIG_REL_ (3.56 ± 2.00 mmol/L) and VIG_LT_ (0.93 ± 0.91 mmol/L). Individual lactate thresholds in relation to the ACSM intensity guidelines are presented in the supplementary materials (ESM1).

**Table 2:** Descriptive characteristics of different sessions

	MOD_ABS_	MOD_REL_	MMOD_LT_	VIG_ABS_	VIG_REL_	VIG_LT_
Speed (km/h)	5.9 ± 0.0	6.9 ± 0.6	5.9 ± 0.6	7.3 ± 0.0	9.2 ± 1.3	7.9 ± 1.3
Speed (%/vPeak)	47.7 ± 6.7	55.5 ± 4.3	47.2 ± 4.3	59.1 ± 8.3	73.1 ± 4.8	62.9 ± 5.3
Distance (km)	3.8 ± 0.0	4.5 ± 0.4	3.8 ± 0.4	2.4 ± 0.0	3.0 ± 0.4	2.6 ± 0.4
Mean HR (%/max)	54.5 ± 7.3	66.6 ± 5.7	56.0 ± 6.4	72.0 ± 8.4	79.7 ± 5.6	70.2 ± 7.4
Peak HR (%/max)	62.3 ± 7.6	74.0 ± 6.7	62.2 ± 7.0	78.6 ± 9.0	86.5 ± 6.8	76.3 ± 8.3
EE (kcal)	281 ± 34	387 ± 59	282 ± 34	231 ± 36	282 ± 40	228 ± 52
%/n < vLT1	77	23	100	8	0	0
%/n vLT1-vLT2	23	77	0	69	31	100
%/n > vLT2	0	0	0	23	69	0

HR = heart rate; EE = energy expenditure during the whole session; %/n < vLT1 = proportion of individuals exercising below the speed of the first lactate threshold; %/n vLT1-vLT2 = proportion of individuals exercising between the speed of the first and second lactate threshold; %/n > vLT2 = proportion of individuals exercising above the speed of the second lactate threshold

### Exercise responses

**Fig. 2:**
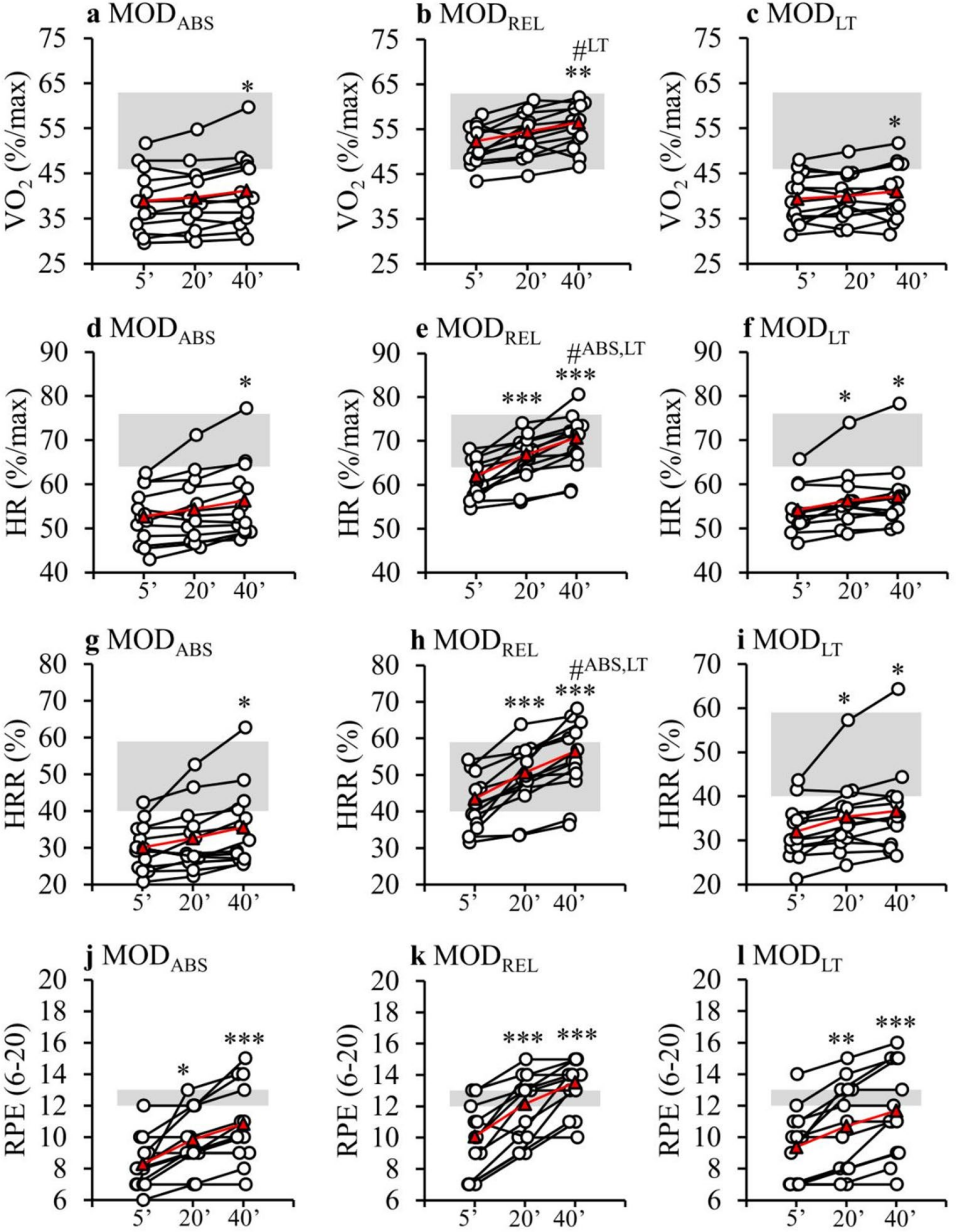
Exercise responses in moderate-intensity sessions that were prescribed based on absolute (ABS), relative (REL), and thresholds-based (LT) intensity domains. The grey-shaded area presents moderate intensity domain and its limits as suggested by ACSM. (**a**) VO_2max_ = maximum oxygen uptake; (**b**) HRmax = maximum heart rate; (**c**) HRR = heart rate reserve; (d) RPE = rating of perceived exertion. ***p < 0.001, **p < 0.01, *p < 0.05 compared to 5’, #*p* < 0.05 between prescription method difference in change from 5’

**Fig. 3:**
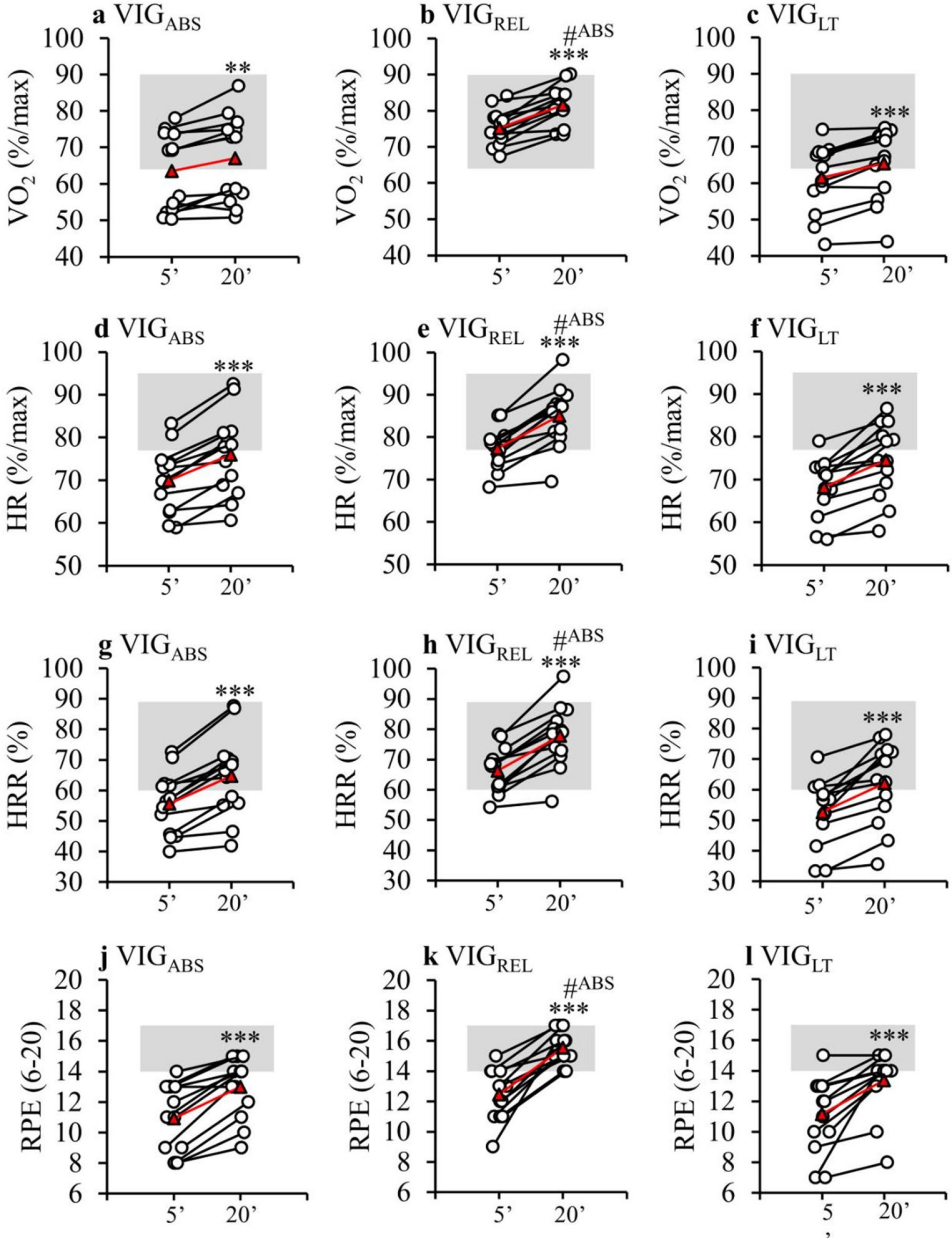
Exercise responses in vigorous-intensity sessions that were prescribed based on absolute (ABS), relative (REL), and thresholds-based (LT) intensity domains. The grey-shaded area presents vigorous intensity domain and its limits as suggested by ACSM. (**a**) VO_2max_ = maximum oxygen uptake; (**b**) HRmax = maximum heart rate; (**c**) HRR = heart rate reserve; (**d**) RPE = rating of perceived exertion. ***p < 0.001, **p < 0.01, *p < 0.05 compared to 5’, #*p* < 0.05 between prescription method difference in change from 5’

Physiological and perceptual changes over time in different exercises and prescription methods are presented in Figs. 2 and 3. A significant main effect of time (*p* < 0.001, partial η² = 0.592–0.870) and prescription method (*p* < 0.05, partial η² = 0.363–0.696) was observed in all parameters in MOD- and VIG-sessions. MOD_REL_ and VIG_REL_ differed (*p* < 0.05) from their counterparts in all other parameters except RPE, where MOD_LT_ was not significantly different (*p* = 0.169) from MOD_REL_.

Time x prescription method interactions were observed in VO_2_ (MOD-sessions *p* = 0.038, partial η² = 0.223; VIG-sessions *p* = 0.038 partial η² = 0.245), HR (MOD-sessions *p* < 0.001, partial η² = 0.686), and HRR (MOD-sessions *p* < 0.001, partial η² = 0.703). Increase in VO_2_ from 5 to 40 min was greater in MOD_REL_ (1.8 ± 1.6 ml/kg/min) than in MOD_LT_ (0.7 ± 0.7 ml/kg/min, *p* = 0.025, ES = 0.71), without significant differences between MOD_REL_ and MOD_ABS_ (0.9 ± 0.9 ml/kg/min, *p* = 0.053, ES = 0.59). In turn, the increase from 5 to 20 min was greater in VIG_REL_ (2.6 ± 1.3 ml/kg/min) than in VIG_ABS_ (1.4 ± 1.2 ml/kg/min, *p* = 0.009, ES = 0.87), without significant differences between VIG_ABS_ and VIG_LT_ (1.6 ± 1.0 ml/kg/min, *p* = 0.075, ES = 0.54). In HR, the increase from 5 to 40 min was greater in MOD_REL_ (16.3 ± 9.5 bpm) than in MOD_LT_ (5.7 ± 7.2 bpm, *p* < 0.001, ES = 2.08) or in MOD_ABS_ (6.6 ± 8.1 bpm, *p* < 0.001, ES = 1.79). Although time x prescription method interaction was not significant (*p* = 0.101, partial η² = 0.174), in pairwise comparisons the increase from 5 to 20 min was greater in VIG_REL_ (14.1 ± 6.2 bpm) than in VIG_ABS_ (11.2 ± 6.4 bpm, *p* = 0.01, ES = 0.85) without significant differences between VIG_REL_ and VIG_LT_ (11.6 ± 7.0 bpm, *p* = 0.107, ES = 0.48). Recovery kinetics of VO_2_ and LnRMSSD during the 10-min recovery are presented in the electronic supplementary materials (ESM2).

### Physiological strain of moderate- and vigorous-intensity exercises

The physiological strain of each session was estimated using several training-load-related metrics. Intensity-related differences within prescription methods are presented in Table 3. EPOC, iTRIMP, blood lactate concentration and sRPE were significantly greater (*p* < 0.001), while LnRMSSD was lower (*p* < 0.001) in REL prescriptions than in ABS or LT prescriptions. In turn, no significant differences were found between ABS and LT prescriptions in any of the metrics.

**Table 3:** Comparison of physiological strain between moderate- and vigorous-intensity sessions within prescription method

	EPOC (L)	LnRMSSD (ms)	iTRIMP (a.u.)	Blood lactate (mmol/L)	sRPE (a.u.)
MOD_ABS_	1.0 ± 0.4	2.9 ± 0.8	14 ± 9	1.0 ± 0.4	86 ± 36
VIG_ABS_	1.7 ± 0.6***	2.2 ± 1.1**	31 ± 21**	2.4 ± 1.5**	54 ± 21**
ES (MOD vs. VIG)	2.01	0.97	0.95	0.87	-1.22
MOD_REL_	1.7 ± 0.5	2.2 ± 0.8	37 ± 15	1.3 ± 0.4	117 ± 34
VIG_REL_	2.5 ± 0.8**	1.5 ± 0.7*	55 ± 25*	4.7 ± 1.8***	89 ± 27**
ES (MOD vs. VIG)	0.90	0.84	0.61	1.92	-1.10
MOD_LT_	1.0 ± 0.3	3.0 ± 0.7	16 ± 12	1.0 ± 0.3	92 ± 53
VIG_LT_	1.5 ± 0.6*	2.1 ± 0.6***	24 ± 12*	2.0 ± 0.8***	58 ± 22*
ES (MOD vs. VIG)	0.69	1.82	0.65	1.64	-0.81

EPOC = excess post exercise oxygen consumption; iTRIMP = individualized training impulse; LnRMSSD = the natural logarithm of root mean square of successive differences; sRPE = session rating of perceived exertion, ES = effect size as Cohen’s d. ***p < 0.001, **p < 0.01, *p < 0.05 compared to MOD

**Fig. 4:**
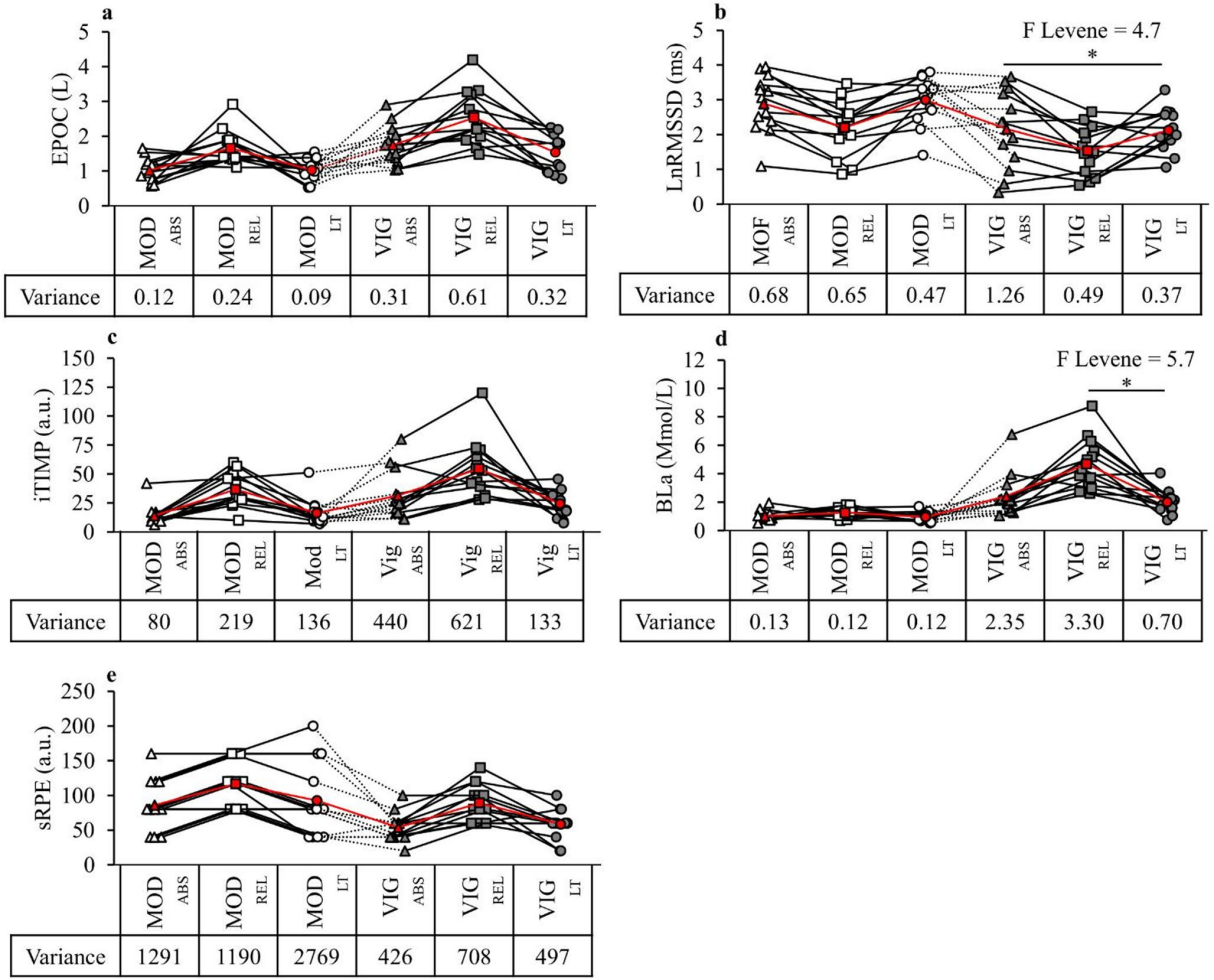
Individual results and variance of training load metrics. *p < 0.05 according to modified Levene’s test. EPOC = excess post exercise oxygen consumption; LnRMSSD = the natural logarithm of root mean square of successive differences iTRIMP = individualized training impulse; BLa = blood lactate; sRPE = session rating of perceived exertion

Individual results and variance regarding training-load-related metrics are presented in Fig. 4. For the moderate-intensity sessions, no significant differences between prescription methods were found in the variance of training-load-related metrics. In turn, within vigorous-intensity sessions, differences were observed between VIG_ABS_ and VIG_LT_ in LnRMSSD (*p* = 0.041), and between VIG_REL_ and VIG_LT_ in blood lactate (*p* = 0.025). In addition to analyzing between-prescription differences in variance, the equivalence was also tested using the TOST procedure. In these analyses, none of the confidence intervals of the compared variables fell entirely within the pre-specified ± 30% equivalence margins (Fig. 5). Therefore, the criteria for statistical equivalence based on p‑values were not met.

**Fig. 5:**
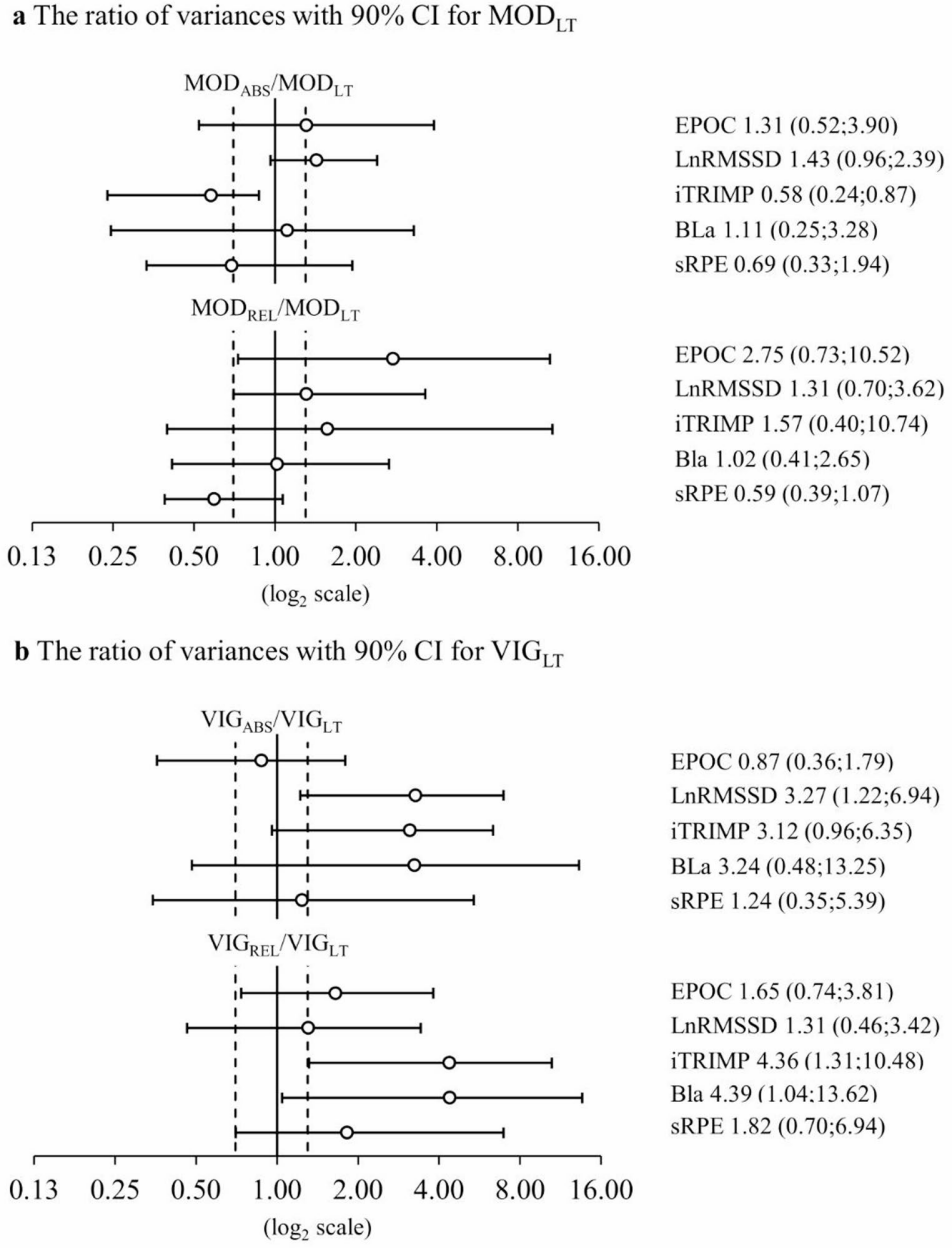
The ratio of variances **(**F-ratio) with 90% confidence intervals (CI) for comparisons between LT and ABS or REL prescriptions. Values above 1.00 indicate greater variance in ABS or REL than LT sessions, while values below 1.00 indicate greater variance in LT than ABS or REL sessions. Dotted line presents the chosen ± 30% limit for equivalent results

### Standardizing doses of moderate- and vigorous-intensity exercises

The general linear mixed-model analysis results are demonstrated for each outcome in the electronic supplemental materials (ESM3). Marginal pseudo R^2^ for different models estimating physiological strain varied from 0.09 (LnRMSSD) to 0.52 (blood lactate) in ABS, from 0.27 (sRPE) to 0.65 (EPOC) in REL, and from 0.16 (sRPE) to 0.65 (blood lactate) in LT. In turn, conditional pseudo R^2^ varied from 0.49 (sRPE) to 0.65 (blood lactate) in ABS, from 0.61 (iTRIMP) to 0.76 (EPOC) in REL, and from 0.58 (sRPE) to 0.78 (blood lactate) in LT. Based on model estimates derived from 78 duration-intensity pairs, Fig. 5 presents intensities that would hypothetically equalize strain between MOD and VIG within each training-load-related metric in 40-min moderate-intensity and 20-min vigorous-intensity exercises. Blood lactate was not involved, because the effect of duration was not significant. Furthermore, ABS prescription did not allow equal strain for any given MOD/VIG-intensity, thus only REL- and LT-based results are presented.

**Fig. 5:**
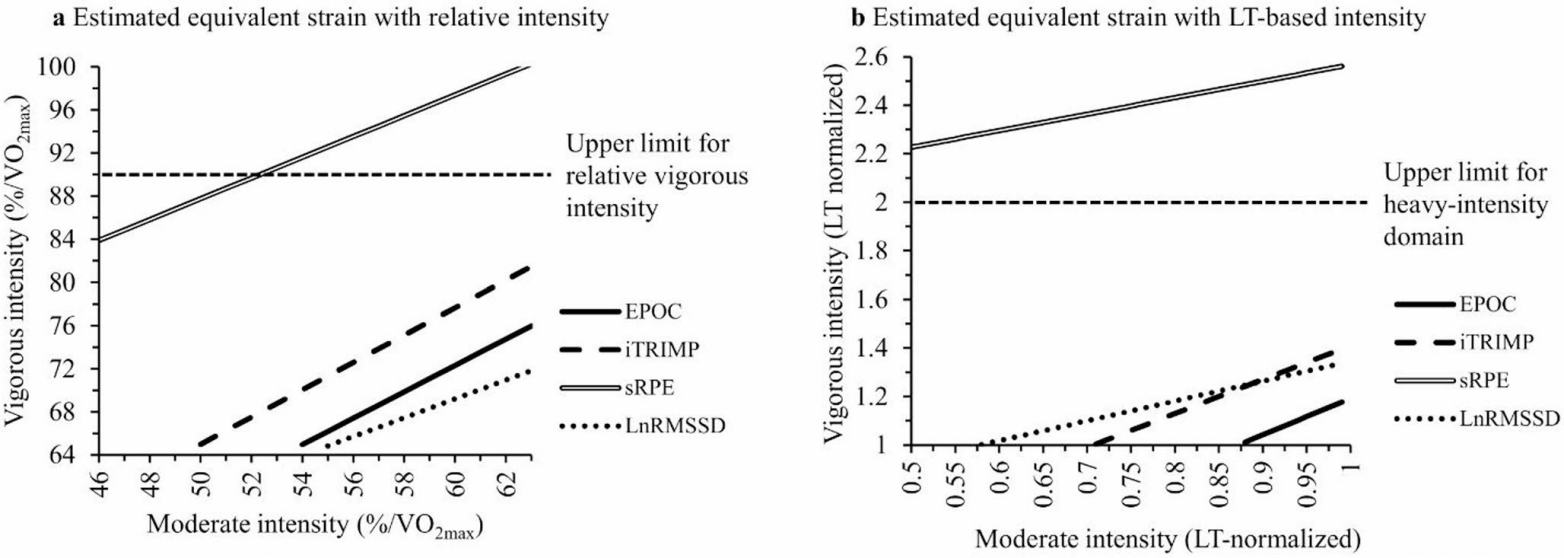
Model-based estimated equivalence lines for 40-min moderate (MOD) and 20-min vigorous (VIG) intensity exercises with relative (**a**) and lactate-threshold-based (**b**) intensity normalizations. If intensity is below the line, strain is greater in MOD, while above the line the strain is greater in VIG. For LT-based normalization 0 = basal VO_2_, 1 = VO_2_ at LT1, 2 = VO_2_ at LT2, 3 = VO_2max_. EPOC = excess post-exercise oxygen consumption; LnRMSSD = the natural logarithm of root mean square of successive differences iTRIMP = individualized training impulse; sRPE = session rating of perceived exertion

## Discussion

Based on most training-load-related metrics, physiological strain was greater during current vigorous-intensity sessions compared to moderate-intensity sessions, regardless of prescription method, when a commonly used 1:2 duration ratio was applied. However, model-based inferences suggested that with appropriate domain intensities, equal strain seems plausible also with the current duration ratio. Variance in blood lactate and HRV responses was lower in VIG_LT_ sessions compared to VIG_REL_ and VIG_ABS_ sessions, respectively, while no such differences were observed in moderate-intensity sessions. Regardless, wide confidence intervals in the ratio of variances indicated a lot of uncertainty in the magnitude of variance across prescription methods.

### Comparison between different intensity prescription methods

Several different exercise intensity determination methods have been proposed in the literature. A common method for exercise prescription is to divide intensity zones (3–5 zones) based on a fixed HR relative to maximum (Schumann et al. 2025). ACSM has also provided target RPE and relative VO_2,_ VO_2_ reserve, and HRR for very light, light, moderate, vigorous, and near maximal to maximal intensity domains (American College of Sports Medicine et al. 2022). While not typically utilized for exercise prescription, absolute thresholds (i.e., MET values) are often used for assessing physical activity volume in population studies and for classifying activities into moderate and vigorous levels (Vähä-Ypyä et al. 2022). A third option, especially used in athletic exercise prescription, is to define intensities based on metabolic thresholds (i.e., lactate or ventilatory) (Jamnick et al. 2020). Importantly, the recent consensus statement by ACSM and Exercise and Sport Science Australia recommended the use of threshold-based approach in both physical activity and exercise prescription contexts (Bishop et al. 2025).

Among the present study population, LT1 varied between light and moderate intensities and LT2 between the upper end of moderate and vigorous intensities, when reflected in relation to the definitions of ACSM. Consequently, REL-exercises were performed at a higher intensity than the ABS or LT exercises. Although the intensity of ABS exercises did not differ significantly from LT exercises, there was substantial inter-individual variability in the intensity domain of the exercise, as determined by threshold-based classification (Table 2). When comparing different intensity prescription methods, a key challenge lies in the ambiguity of interpreting threshold-based domains in relation to absolute or relative intensity domains, since the latter are quite broad and lack the same physiological justification as the threshold-based domains. In the aforementioned consensus statement (Bishop et al. 2025), the heavy-intensity domain was considered equivalent to the moderate intensity, while the severe-intensity domain corresponded to vigorous intensity. In turn, MacIntosh et al. (2021) regarded threshold-based and relative moderate-intensity domains as surrogates, which highlights the lack of consensus regarding the physiological rationale and target of relative domains. In the current study population, the division proposed by Bishop et al. (2025) seemed quite appropriate, but in more trained populations, the division of MacIntosh et al. (2021) might be more accurate (Nuuttila et al. 2025).

As anticipated, the physiological responses elicited by ABS, REL, and LT exercises were aligned with previous literature and associated with intensity domains (Jones et al. 2011; Jamnick et al. 2020): The increase in VO_2_ was greater during MOD_REL_ than MOD_LT_, and differences were also found between VIG_ABS_ and VIG_REL_. The first result is most likely attributable to the fact that during the MOD_REL_ session, most individuals exercised within the heavy-intensity domain, characterized by the presence of the VO₂ slow component, while MOD_LT_ was performed in the moderate-intensity domain. Conversely, in the VIG_REL_ session, some participants had already reached the severe-intensity domain, with no evident stabilization in VO₂, while some individuals in the VIG_ABS_ session were still exercising at the moderate-intensity domain. Blood lactate responses were also consistent with literature across the different exercises: MOD_LT_ slightly decreased blood lactate concentration, while no systematic changes were observed in MOD_ABS_ or MOD_REL_. VIG_REL_ resulted in a greater increase than VIG_ABS_ or VIG_LT_, and especially among participants in the severe-intensity domain, the rise was pronounced. Conclusively, the responses appeared to reflect well the phenomena for which different intensity prescription methods have been either criticized or commended (Vähä-Ypyä et al. 2022; Hansen et al. 2025), thereby lending support to the intensity choices across different prescription methods of the present study.

### Magnitude and heterogeneity of physiological strain

Regarding the magnitude of physiological strain, all physiological training-load-related metrics suggested that current vigorous-intensity exercises were more demanding than moderate-intensity exercises regardless of the prescription method. Interestingly, the subjective sRPE was the only metric indicating that moderate-intensity sessions could be more demanding than the vigorous-intensity ones. This observation can at least partly relate to the different nature of the parameter when compared to other metrics. While EPOC, for instance, increases somewhat linearly with time, the session-RPE increases quadratically (Matomäki et al. 2024): the duration is the other multiplier in the metric itself, and it also affects the perceived exertion. Therefore, it might overestimate the effect of exercise duration on physiological strain, as observed in the present study. Of note, metrics incorporating arbitrary time component may be limited by the fact that the relationship between exercise duration and physiological strain is not necessarily straightforward (Hofmann and Tschakert 2017) or uniform between individuals (i.e., effects of physiological resilience) (Jones 2023). To obtain a more precise assessment of subjective strain, it may be justified to use more extensive questionnaires, such as NASA task load index which was recently reported to correlate with exercise-induced performance decrement better than physiological metrics (D’Alleva et al. 2025). Although current vigorous-intensity sessions seemed more demanding than moderate-intensity sessions, the contribution of intensity within a domain should also be acknowledged. As demonstrated with modeled estimates in Fig. 5, with a combination of sufficiently high moderate intensity and low vigorous intensity, it seems plausible to equalize strain between these sessions with 1:2 duration ratio. At the same time, these analyses demonstrated the limitations of ABS-prescription, as it was not possible to standardize moderate and vigorous exercise doses according to physiological strain in the current sample.

Heterogeneity of acute exercise responses (and physiological strain) after different intensity prescription methods has mainly been examined through changes in blood lactate (Lansley et al. 2011; Meyler et al. 2023; Pacitti et al. 2025), and the large heterogeneity of blood lactate responses of fixed intensity anchors has been emphasized for a while now (Meyer et al. 1999; Scharhag-Rosenberger et al. 2010). Interestingly, recent studies of Meyler et al. (2023) and Pacitti et al. (2025) found no differences in the variance of blood lactate responses in comparison between exercises fixed to maximum anchors or individual thresholds. Similarly, in the current study, there were no differences in the variance of blood lactate responses or any other training-load -related metric within moderate-intensity exercises. In turn, the variance was significantly greater after VIG_REL_ than after VIG_LT_. The lack of differences in the moderate-intensity exercises most likely relates to the fact that the intensity was relatively low in all sessions, which induced only minor increases in blood lactate. This also raises the question of whether the method of intensity prescription has that meaningful impact at low intensities, where the resulting physiological strain is inherently relatively modest. While Meyler et al. (2023) used similar fixed intensities (55 and 75%/VO_2max_) that were used in the current study, the differences regarding the blood lactate responses to vigorous-intensity exercise could relate to the threshold method applied, as they used critical power for the upper threshold instead of lactate threshold. Although critical power has been suggested to reflect similar physiological processes as the maximal metabolic steady state, the applied method can modify this connection (Caen et al. 2024). Another notion compared to previous studies is that they have utilized cycling as a training mode (Lansley et al. 2011; Meyler et al. 2023; Pacitti et al. 2025), while results on walking and running are limited. Therefore, the possible effect of training mode on observed responses and their heterogeneity cannot be ruled out.

Despite the differences in blood lactate responses and their variance between VIG_REL_ and VIG_LT_, there were no differences in the variance of other markers of physiological strain between these sessions. Although many studies have related their findings to blood lactate, it is worth noting that the physiological meaning and functioning of lactate has been widely discussed in recent years (Brooks et al. 2023; De Wit 2025). Judging by the blood lactate variance, it could be expected that iTRIMP (based on an individual lactate profile (Manzi et al. 2009) would also differ accordingly. A trend in this direction was observed but the lack of statistically significant differences was probably related to day-to-day variations in HR (Lamberts and Lambert 2009), and therefore, the HR–lactate relationship during the exercises does not correspond exactly to the ratio determined in the incremental test. In addition to blood lactate responses, the only other statistically significant difference was observed between the variance of post-exercise HRV in VIG_ABS_ vs. VIG_LT_. It is known that HRV recovery is affected by the intensity more than the duration of preceding exercise (Seiler et al. 2007; Kaikkonen et al. 2010; Nuuttila et al. 2020). Thus, the greater variance was somewhat expected, because in VIG_ABS_ the individuals were distributed to all three intensity domains (moderate, heavy, severe). The lack of difference compared to VIG_REL_ possibly relates to the nature of HRV. While other training load metrics increase with greater physiological strain, HRV decreases (Kaikkonen et al. 2010), and consequently, the inter-individual variance is likely to decrease at the same time. On that account, acute post-exercise HRV might have limited ability to differentiate strain within vigorous-intensity or heavy-to-severe intensity domains.

Although only a few significant differences were found in the variance of training-load-related metrics, results did not necessarily indicate equal variances between prescription methods. This was demonstrated by the equivalence tests that did not find equivalent variance between LT and ABS or REL prescriptions in any of the outcomes. Even though variances in VIG sessions seemed smaller with LT prescription, wide confidence intervals highlighted a lot of uncertainty in these results that were probably attributable to the relatively small sample size.

### Implications and future perspectives

The current study aimed to examine how physiological strain could be equalized between intensity domains using different intensity–duration combinations. These findings should be considered preliminary rather than direct empirical demonstrations due to the relatively limited amount of data, and therefore, larger scale studies are needed to refine the estimates. Since there is no single gold-standard method for measuring physiological strain, it is challenging to determine the preferred outcome for standardizing the exercise dose (Matomäki et al. 2024). Moreover, the current study demonstrated differences between metrics, specifically between sRPE and other metrics. Thus, identifying the most valid metrics reflecting the physiological strain (and adaptive signal) of a given exercise would be important for accurately designing effective interventions.

There is a clear rationale for the threshold-based exercise prescription in terms of standardizing physiological strain (Iannetta et al. 2020; Jamnick et al. 2020). The recent consensus statement (Bishop et al. 2025) of ACSM and ESSA also recommended the use of metabolic thresholds for intensity prescription, which will likely encourage wider application of thresholds. However, as shown in the present study, even threshold-based methods induce considerable inter-individual variance in physiological responses, highlighting how individuals respond differently to standardized exercise stimuli. Therefore, it is crucial to identify underlying factors that may contribute to differences in responses and consider how this information could be used when designing appropriate exercise doses for an individual. Furthermore, there is a need to identify simple, yet accurate methods to assess intensity domains across individuals. Ultimately, future work should also compare prescription methods that are commended by experts of the field (e.g., LT vs. VT vs. critical power/speed).

### Limitations

The sample of the study was fairly small and limited to habitually active males. Larger dataset would have increased the statistical power of the analyses, and in a more heterogeneous sample (fitness level, age, sex), inter-individual differences in responses (e.g., ABS sessions) might have emerged more clearly. On the other hand, the sample size of the study was comparable to that of previous studies on the topic (Lansley et al. 2011; Meyler et al. 2023; Pacitti et al. 2025) whose findings this research complemented with new perspectives. The current study focused solely on men, but in the future, it will be important to investigate similar aspects in women as well. Especially, since there seems to be sex-related differences in the thresholds in relation to maximum values (Benítez-Muñoz et al. 2024; Nuuttila et al. 2025). Lastly, the current study examined only a limited number of intensity-duration pairs; thus, the results (e.g., model-derived estimates of equal MOD/VIG-strain) cannot be directly transferred to different combinations. Despite these limitations, the current study provided novel exploratory results on physiological strain and the effects of applied prescription methods in a well-controlled randomized cross-over research setting.

## Conclusions

In conclusion, the physiological strain of vigorous-intensity exercises seemed greater than that of moderate-intensity exercises, when a commonly used 1:2 ratio in duration and halfway intensity within a domain was applied. However, with suitable within-domain intensities, equal strain seems plausible also with the current duration ratio based on modeled estimates. Observed differences in the variance of blood lactate and HRV responses after vigorous-intensity exercises suggest that an appropriate prescription method for standardizing physiological strain might become increasingly important at higher exercise intensities. Nevertheless, wide confidence intervals in the ratio of variances indicated a lot of uncertainty in the magnitude of variance and a need for a large-scale studies on this topic for more robust conclusions.

## Supplementary Information

Below is the link to the electronic supplementary material.

Supplementary Material 1

Supplementary Material 2

Supplementary Material 3

## Data Availability

The datasets generated and/or analyzed during the current study are available from the corresponding author on reasonable request.

## Abbreviations

EPOC: Excess post exercise oxygen consumption
HR: Heart rate
HRR: Heart rate reserve
HRV: Heart rate variability
iTRIMP: Individualized training impulse
LnRMSSD: The natural logarithm of the root mean square of successive differences
LT1: The first lactate threshold
LT2: The second lactate threshold
MET: Metabolic equivalent
MOD: Moderate intensity
MOD_ABS_: Moderate-intensity session prescribed by absolute metabolic equivalents
MOD_REL_: Moderate-intensity session prescribed by relative maximum oxygen uptake
MOD_LT_: Moderate-intensity session prescribed by lactate thresholds
RPE: Rating of perceived exertion
sRPE: Session rating of perceived exertion
VIG: Vigorous intensity
VIG_ABS_: Vigorous-intensity session prescribed by absolute metabolic equivalents
VIG_REL_: Vigorous-intensity session prescribed by relative maximum oxygen uptake
VIG_LT_: Vigorous-intensity session prescribed by lactate thresholds
VO_2_: Oxygen uptake
VO_2max_: Maximum oxygen uptake
vPeak: Maximum treadmill test speed

## References

[CR1] Al Haddad H, Laursen P, Chollet D et al (2011) Reliability of resting and postexercise heart rate measures. Int J Sports Med 32:598–605. 10.1055/s-0031-1275356 21574126 10.1055/s-0031-1275356

[CR2] American College of Sports Medicine (ACSM) (2022) ACSM’s guidelines for exercise testing and prescription, eleventh edition. Wolters Kluwer, Philadelphia, PA. ISBN 978-1975150181.

[CR3] Banister E (1991) Modeling elite athletic performance. In: Physiological Testing of Elite Athletes. pp 403–424

[CR4] Benítez-Muñoz JA, Rojo-Tirado MÁ, Benito Peinado PJ et al (2024) Greater relative first and second lactate thresholds in females compared with males: consideration for exercise prescription. Int J Sports Physiol Perform 1–7. 10.1123/ijspp.2024-0079 10.1123/ijspp.2024-0079 39467538

[CR5] Bentley DJ, Newell J, Bishop D (2007) Incremental exercise test design and analysis: implications for performance diagnostics in endurance athletes. Sports Med 37:575–586. 10.2165/00007256-200737070-00002 17595153 10.2165/00007256-200737070-00002

[CR6] Bishop DJ, Beck B, Biddle SJH et al (2025) Physical activity and exercise intensity terminology: A joint American college of sports medicine (ACSM) expert statement and exercise and sport science Australia (ESSA) consensus statement. Med Sci Sports Exerc 57:2599–2613. 10.1249/MSS.0000000000003795 41085254 10.1249/MSS.0000000000003795

[CR7] Borg GA (1982) Psychophysical bases of perceived exertion. Med Sci Sports Exerc 14:377–381 7154893

[CR8] Borresen J, Ian Lambert M (2009) The quantification of training Load, the training response and the effect on performance. Sports Med 39:779–795. 10.2165/11317780-000000000-00000 19691366 10.2165/11317780-000000000-00000

[CR9] Brooks GA, Osmond AD, Arevalo JA et al (2023) Lactate as a myokine and exerkine: drivers and signals of physiology and metabolism. J Appl Physiol 134:529–548. 10.1152/japplphysiol.00497.2022 36633863 10.1152/japplphysiol.00497.2022 PMC9970662

[CR10] Brown MB, Forsythe AB (1974) Robust tests for the equality of variances. J Am Stat Assoc 69:364–367. 10.1080/01621459.1974.10482955

[CR11] Bull FC, Al-Ansari SS, Biddle S et al (2020) World health organization 2020 guidelines on physical activity and sedentary behaviour. Br J Sports Med 54:1451–1462. 10.1136/bjsports-2020-102955 33239350 10.1136/bjsports-2020-102955 PMC7719906

[CR12] Caen K, Poole DC, Vanhatalo A, Jones AM (2024) Critical power and maximal lactate steady state in cycling: watts the difference? Sports Med 54:2497–2513. 10.1007/s40279-024-02075-4 39196486 10.1007/s40279-024-02075-4

[CR14] Coates AM, Joyner MJ, Little JP et al (2023) A perspective on High-Intensity interval training for performance and health. Sports Med 53:85–96. 10.1007/s40279-023-01938-6 37804419 10.1007/s40279-023-01938-6 PMC10721680

[CR15] D’Alleva M, Nicolò A, Bot F et al (2025) The relationship between training load and acute performance decrements following different types of training sessions in Well-Trained runners. Int J Sports Physiol Perform 20:823–833. 10.1123/ijspp.2024-0453 40280552 10.1123/ijspp.2024-0453

[CR16] De Wit C (2025) Lactate refurbished: cardiovascular support during metabolic stress and fuel rather than waste. Acta Physiol 241:e70064. 10.1111/apha.70064 10.1111/apha.70064 40432421

[CR17] Foster C, Daines E, Hector L et al (1996) Athletic performance in relation to training load. Wis Med J 95:370–374 8693756

[CR18] Hansen D, Junior GC, Milani JGPO et al (2025) Advancing aerobic exercise training intensity prescription in health and disease beyond standard recommendations: A call to action. Sports Med. 10.1007/s40279-025-02272-9 40613962 10.1007/s40279-025-02272-9

[CR19] Herrmann SD, Willis EA, Ainsworth BE et al (2024) 2024 adult compendium of physical activities: A third update of the energy costs of human activities. J Sport Health Sci 13:6–12. 10.1016/j.jshs.2023.10.010 38242596 10.1016/j.jshs.2023.10.010 PMC10818145

[CR20] Hilz MJ, Dütsch M (2006) Quantitative studies of autonomic function. Muscle Nerve 33:6–20. 10.1002/mus.20365 15965941 10.1002/mus.20365

[CR21] Hofmann P, Tschakert G (2017) Intensity- and Duration-Based options to regulate endurance training. Front Physiol 8:337. 10.3389/fphys.2017.00337 28596738 10.3389/fphys.2017.00337 PMC5442222

[CR22] Iannetta D, Inglis EC, Mattu AT et al (2020) A critical evaluation of current methods for exercise prescription in women and men. Med Sci Sports Exerc 52:466–473. 10.1249/MSS.0000000000002147 31479001 10.1249/MSS.0000000000002147

[CR23] Iannetta D, Keir DA, Fontana FY et al (2021) Evaluating the accuracy of using fixed ranges of METs to categorize exertional intensity in a heterogeneous group of healthy individuals: implications for cardiorespiratory fitness and health outcomes. Sports Med 51:2411–2421. 10.1007/s40279-021-01476-z 33900580 10.1007/s40279-021-01476-z

[CR24] Impellizzeri FM, Shrier I, McLaren SJ et al (2023) Understanding training load as exposure and dose. Sports Med 53:1667–1679. 10.1007/s40279-023-01833-0 37022589 10.1007/s40279-023-01833-0 PMC10432367

[CR25] Jamnick NA, Pettitt RW, Granata C et al (2020) An examination and critique of current methods to determine exercise intensity. Sports Med 50:1729–1756. 10.1007/s40279-020-01322-8 32729096 10.1007/s40279-020-01322-8

[CR26] Jones AM (2023) The fourth dimension: physiological resilience as an independent determinant of endurance exercise performance. J Physiol JP284205. 10.1113/JP284205 10.1113/JP284205 37606604

[CR27] Jones AM, Grassi B, Christensen PM et al (2011) Slow component of V˙O2 kinetics: mechanistic bases and practical applications. Med Sci Sports Exerc 43:2046–2062. 10.1249/MSS.0b013e31821fcfc1 21552162 10.1249/MSS.0b013e31821fcfc1

[CR28] Kaikkonen P, Hynynen E, Mann T et al (2010) Can HRV be used to evaluate training load in constant load exercises? Eur J Appl Physiol 108:435–442. 10.1007/s00421-009-1240-1 19826833 10.1007/s00421-009-1240-1

[CR29] Keskinen K, Häkkinen K, Kallinen M (2018) Fyysisen Kunnon mittaaminen: käsi- Ja Oppikirja Kuntotestaajille. Liikuntatieteellinen Seura

[CR30] Krejčí J, Botek M, McKune AJ (2018) Stabilization period before capturing an ultra-short vagal index can be shortened to 60 s in endurance athletes and to 90 s in university students. PLoS ONE 13:e0205115. 10.1371/journal.pone.0205115 30296274 10.1371/journal.pone.0205115 PMC6175275

[CR31] Lamberts RP, Lambert MI (2009) Day-to-Day variation in heart rate at different levels of submaximal exertion: implications for monitoring training. J Strength Conditioning Res 23:1005–1010. 10.1519/JSC.0b013e3181a2dcdc 10.1519/JSC.0b013e3181a2dcdc 19387374

[CR32] Lansley KE, DiMenna FJ, Bailey SJ, Jones AM (2011) A ‘New’ method to normalise exercise intensity. Int J Sports Med 32:535–541. 10.1055/s-0031-1273754 21563028 10.1055/s-0031-1273754

[CR33] Lipponen JA, Tarvainen MP (2019) A robust algorithm for heart rate variability time series artefact correction using novel beat classification. J Med Eng Technol 43:173–181. 10.1080/03091902.2019.1640306 31314618 10.1080/03091902.2019.1640306

[CR34] Lucia A, Hoyos J, Santalla A et al (2003) Tour de France versus Vuelta a Espa??a: which is harder? Med Sci Sports Exerc 35:872–878. 10.1249/01.MSS.0000064999.82036.B4 12750600 10.1249/01.MSS.0000064999.82036.B4

[CR35] MacIntosh BR, Murias JM, Keir DA, Weir JM (2021) What is moderate to vigorous exercise intensity? Front Physiol 12:682233. 10.3389/fphys.2021.682233 34630133 10.3389/fphys.2021.682233 PMC8493117

[CR36] Mann T, Lamberts R, Lambert M (2014a) Day-to-day variation in heart rate recovery and excess post-exercise oxygen consumption after a submaximal treadmill protocol. Int SportMed J 15:352–364

[CR37] Mann TN, Webster C, Lamberts RP, Lambert MI (2014b) Effect of exercise intensity on post-exercise oxygen consumption and heart rate recovery. Eur J Appl Physiol 114:1809–1820. 10.1007/s00421-014-2907-9 24878688 10.1007/s00421-014-2907-9

[CR38] Manzi V, Iellamo F, Impellizzeri F et al (2009) Relation between individualized training impulses and performance in distance runners. Med Sci Sports Exerc 41:2090–2096. 10.1249/MSS.0b013e3181a6a959 19812506 10.1249/MSS.0b013e3181a6a959

[CR39] Matomäki P, Nuuttila O-P, Heinonen OJ et al (2024) How to equalize High- and Low-Intensity endurance exercise dose. Int J Sports Physiol Perform 19:851–859. 10.1123/ijspp.2024-0015 39032919 10.1123/ijspp.2024-0015

[CR40] Meyer T, Gabriel HHW, Kindermann W (1999) Is determination of exercise intensities as percentages of &OV0312O2max or HRmax adequate? Med Sci Sports Exerc 31:1342–1345. 10.1097/00005768-199909000-00017 10487378 10.1097/00005768-199909000-00017

[CR41] Meyler S, Bottoms L, Muniz-Pumares D (2021) Biological and methodological factors affecting response variability to endurance training and the influence of exercise intensity prescription. Exp Physiol 106:1410–1424. 10.1113/EP089565 34036650 10.1113/EP089565

[CR42] Meyler S, Bottoms L, Wellsted D, Muniz-Pumares D (2023) Variability in exercise tolerance and physiological responses to exercise prescribed relative to physiological thresholds and to maximum oxygen uptake. Exp Physiol 108:581–594. 10.1113/EP090878 36710454 10.1113/EP090878 PMC10103872

[CR44] Mujika I (2017) Quantification of training and competition loads in endurance sports: methods and applications. Int J Sports Physiol Perform 12. 10.1123/ijspp.2016-0403. S2-9-S2-17 10.1123/ijspp.2016-0403 27918666

[CR45] Norha J, Sjöros T, Garthwaite T et al (2023) Effects of reducing sedentary behavior on cardiorespiratory fitness in adults with metabolic syndrome: A 6-month RCT. Scandinavian Med Sci Sports 33:1452–1461. 10.1111/sms.14371 10.1111/sms.14371 37073456

[CR47] Nuuttila O-P, Kyröläinen H, Häkkinen K, Nummela A (2020) Acute physiological responses to four running sessions performed at different intensity zones. Int J Sports Med A. 10.1055/a-1263-1034. -1263-1034 10.1055/a-1263-1034 33176386

[CR48] Nuuttila O-P, Kyröläinen H, Kokkonen V-P, Uusitalo A (2024) Morning versus nocturnal heart rate and heart rate variability responses to intensified training in recreational runners. Sports Med - Open 10:120. 10.1186/s40798-024-00779-5 39503915 10.1186/s40798-024-00779-5 PMC11541970

[CR46] Nuuttila O-P, Kaikkonen P, Sievänen H et al (2025) The accuracy of fixed intensity anchors to estimate lactate thresholds in recreational runners. Eur J Appl Physiol. 10.1007/s00421-025-05748-8 40088270 10.1007/s00421-025-05748-8 PMC12354492

[CR49] Pacitti LJ, Shikaze KE, Simpson-Stairs N et al (2025) Individual variability in lactate response to cycling prescribed using physiological thresholds and peak work rate: a crossover within-participant repeated measures study. Eur J Appl Physiol. 10.1007/s00421-025-05711-7 39907774 10.1007/s00421-025-05711-7

[CR50] Passfield L, Murias JM, Sacchetti M, Nicolò A (2022) Validity of the Training-Load concept. Int J Sports Physiol Perform 17:507–514. 10.1123/ijspp.2021-0536 35247874 10.1123/ijspp.2021-0536

[CR51] Penttilä J, Helminen A, Jartti T et al (2001) Time domain, geometrical and frequency domain analysis of cardiac vagal outflow: effects of various respiratory patterns. Clin Physiol 21:365–376. 10.1046/j.1365-2281.2001.00337.x 11380537 10.1046/j.1365-2281.2001.00337.x

[CR52] Piercy KL, Troiano RP, Ballard RM et al (2018) The Physical Activity Guidelines for Americans. JAMA 320:2020. 10.1001/jama.2018.14854 10.1001/jama.2018.14854 PMC9582631 30418471

[CR53] Sanders D, Abt G, Hesselink MKC et al (2017) Methods of monitoring training load and their relationships to changes in fitness and performance in competitive road cyclists. Int J Sports Physiol Perform 12:668–675. 10.1123/ijspp.2016-0454 28095061 10.1123/ijspp.2016-0454

[CR54] Scharhag-Rosenberger F, Meyer T, Gäßler N et al (2010) Exercise at given percentages of VO2max: heterogeneous metabolic responses between individuals. J Sci Med Sport 13:74–79. 10.1016/j.jsams.2008.12.626 19230766 10.1016/j.jsams.2008.12.626

[CR55] Schaun GZ (2017) The maximal oxygen uptake verification phase: a light at the end of the tunnel? Sports Med - Open 3:44. 10.1186/s40798-017-0112-1 29218470 10.1186/s40798-017-0112-1 PMC5721097

[CR56] Schumann M, Feuerbacher JF, Heinrich L et al (2025) Using Free-Living heart rate data as an objective method to assess physical activity: A scoping review and recommendations by the INTERLIVE-Network targeting consumer wearables. Sports Med 55:275–300. 10.1007/s40279-024-02159-1 39893599 10.1007/s40279-024-02159-1 PMC11946962

[CR57] Seiler S, Haugen O, Kuffel E (2007) Autonomic recovery after exercise in trained athletes: intensity and duration effects. Med Sci Sports Exerc 39:1366–1373. 10.1249/mss.0b013e318060f17d 17762370 10.1249/mss.0b013e318060f17d

[CR58] Shvartz E, Reibold RC (1990) Aerobic fitness norms for males and females aged 6 to 75 years: a review. Aviat Space Environ Med 61:3–11 2405832

[CR59] Uusitalo ALT, Tahvanainen KUO, Uusitalo AJ, Rusko HK (1996) Non-invasive evaluation of sympathovagal balance in athletes by time and frequency domain analyses of heart rate and blood pressure variability. Clin Physiol 16:575–588. 10.1111/j.1475-097X.1996.tb00735.x 8937797 10.1111/j.1475-097x.1996.tb00735.x

[CR61] Vähä-Ypyä H, Vasankari T, Husu P et al (2015) Validation of Cut-Points for evaluating the intensity of physical activity with Accelerometry-Based mean amplitude deviation (MAD). PLoS ONE 10:e0134813. 10.1371/journal.pone.0134813 26292225 10.1371/journal.pone.0134813 PMC4546343

[CR60] Vähä-Ypyä H, Sievänen H, Husu P et al (2022) How adherence to the updated physical activity guidelines should be assessed with accelerometer? Eur J Pub Health 32:i50–i55. 10.1093/eurpub/ckac078 36031824 10.1093/eurpub/ckac078 PMC9421411

[CR62] Wallace LK, Slattery KM, Coutts AJ (2014a) A comparison of methods for quantifying training load: relationships between modelled and actual training responses. Eur J Appl Physiol 114:11–20. 10.1007/s00421-013-2745-1 24104194 10.1007/s00421-013-2745-1

[CR63] Wallace LK, Slattery KM, Impellizzeri FM, Coutts AJ (2014b) Establishing the criterion validity and reliability of common methods for quantifying training load. J Strength Conditioning Res 28:2330–2337. 10.1519/JSC.0000000000000416 10.1519/JSC.0000000000000416 24662229

[CR64] Wasfy MM, Baggish AL (2016) Exercise dose in clinical practice. Circulation 133:2297–2313. 10.1161/CIRCULATIONAHA.116.018093 27267537 10.1161/CIRCULATIONAHA.116.018093 PMC4902280

