# Supplementary material for: Standardizing moderate- and vigorous-intensity exercise doses by physiological strain: an exploratory randomized cross-over study
Source: Eur J Appl Physiol. 2026 Mar 9;126(7):3637–53. doi: 10.1007/s00421-026-06157-1 (PMC13380570; doi:10.1007/s00421-026-06157-1)
Supplement: Supplementary file 1 — Supplementary Material 1 [file 421_2026_6157_MOESM1_ESM.docx]

Standardizing moderate- and vigorous-intensity exercise doses by physiological strain: an exploratory randomized cross-over study

Olli-Pekka Nuuttila^1,2*^, Piia Kaikkonen^,3^, Timi Malinen^2^, Harri Sievänen^1^, Tommi Vasankari^1,4^Heikki Kyröläinen^2^

1 The UKK Institute for Health Promotion Research, Finland

2 Faculty of Sport and Health Sciences, University of Jyväskylä, Finland

3 Tampere Research Center of Sports Medicine, UKK Institute, Finland

4 Faculty of Medicine and Health Technology, Tampere University, Finland

**Corresponding author:**

Olli-Pekka Nuuttila, E-mail: [olli-pekka.nuuttila@ukkinstituutti.fi](mailto:olli-pekka.nuuttila@ukkinstituutti.fi)


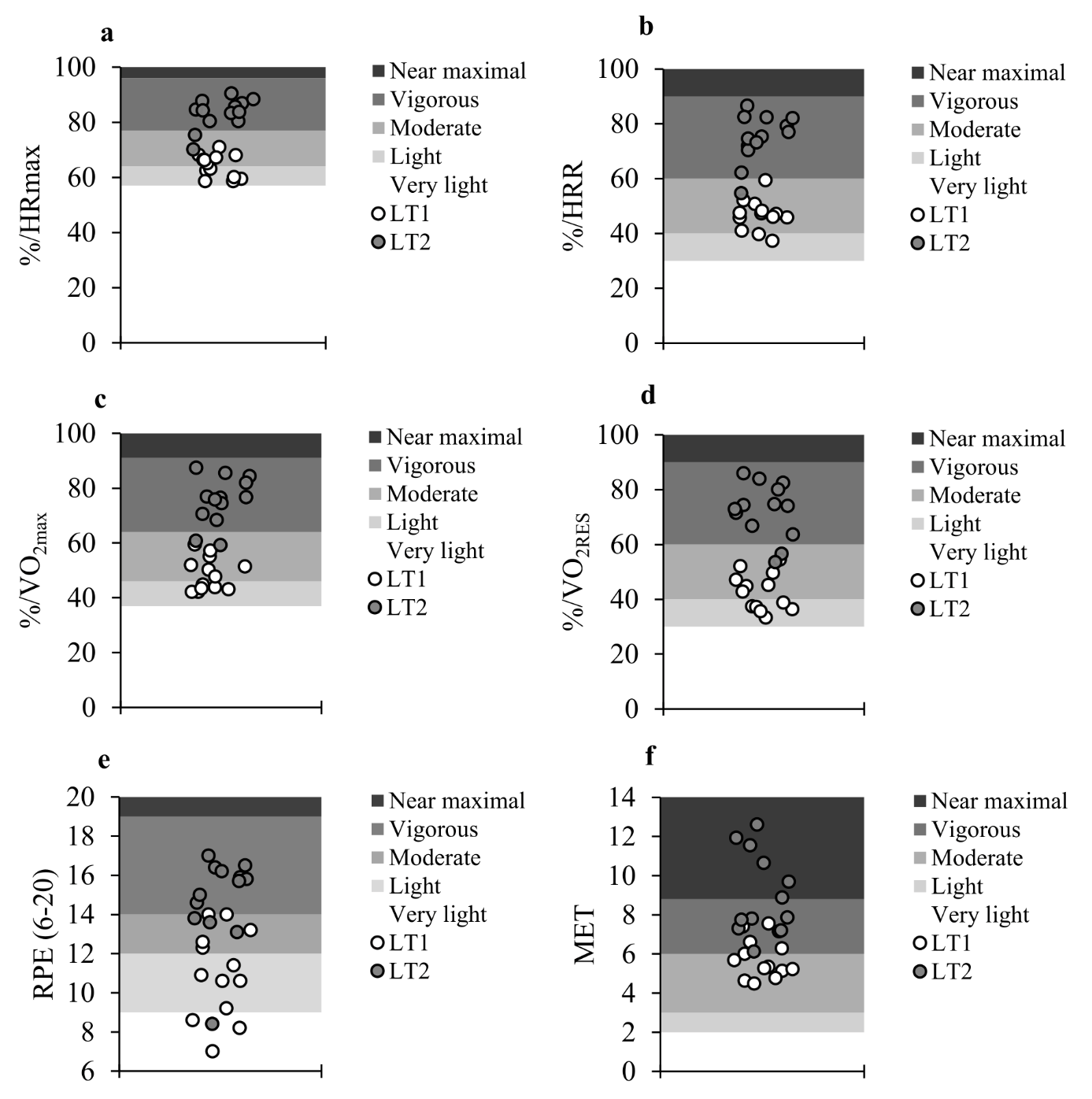
**Electronic supplementary material 1.** The first (LT1) and second (LT2) lactate thresholds in relation to the intensity domains proposed by American College of Sports Medicine (1). HRmax = maximum heart rate (a); HRR = heart rate reserve (b); VO2max = maximum oxygen uptake (c); VO2res = oxygen uptake reserve (d); RPE = rating of perceived exertion (e); MET = metabolic equivalents (f).

**References:**

1. ACSM’s guidelines for exercise testing and prescription. American College of Sports Medicine. 12th ed. Philadelphia (PA): Wolters Kluwer, 2025.
