# Supplementary material for: Standardizing moderate- and vigorous-intensity exercise doses by physiological strain: an exploratory randomized cross-over study
Source: Eur J Appl Physiol. 2026 Mar 9;126(7):3637–53. doi: 10.1007/s00421-026-06157-1 (PMC13380570; doi:10.1007/s00421-026-06157-1)
Supplement: Supplementary file 2 — Supplementary Material 2 [file 421_2026_6157_MOESM2_ESM.docx]

Standardizing moderate- and vigorous-intensity exercise doses by physiological strain: an exploratory randomized cross-over study

Olli-Pekka Nuuttila^1,2*^, Piia Kaikkonen^,3^, Timi Malinen^2^, Harri Sievänen^1^, Tommi Vasankari^1,4^Heikki Kyröläinen^2^

1 The UKK Institute for Health Promotion Research, Finland

2 Faculty of Sport and Health Sciences, University of Jyväskylä, Finland

3 Tampere Research Center of Sports Medicine, UKK Institute, Finland

4 Faculty of Medicine and Health Technology, Tampere University, Finland

**Corresponding author:**

Olli-Pekka Nuuttila, E-mail: [olli-pekka.nuuttila@ukkinstituutti.fi](mailto:olli-pekka.nuuttila@ukkinstituutti.fi)


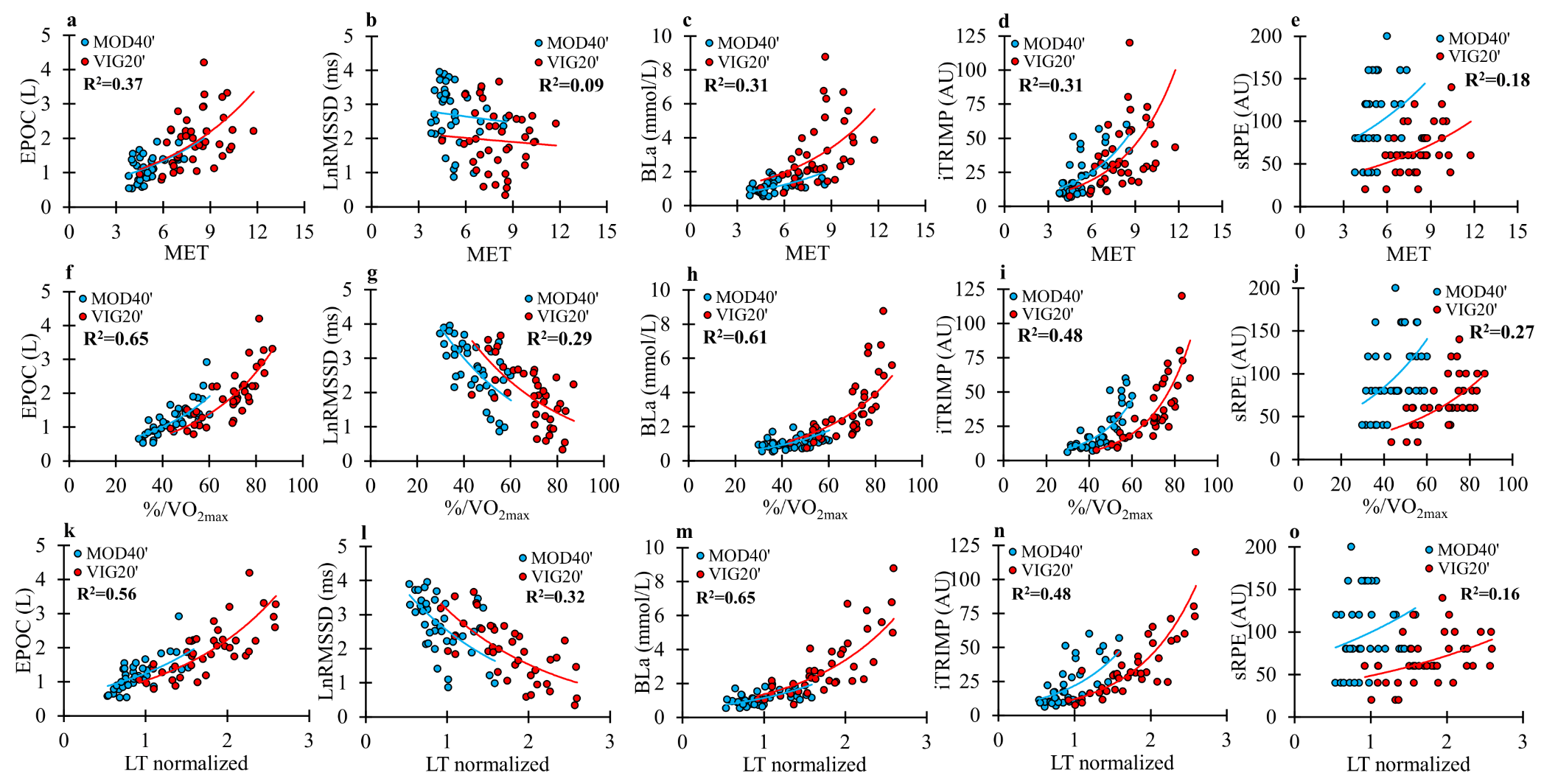
**Electronic supplementary material 2.** Predicted physiological strain in 40-min moderate-intensity (MOD = blue line) and 20-min vigorous-intensity (VIG = red line) exercises derived from the fixed-effect estimates of the generalized linear mixed models. Results are shown together with the measured data points (6 sessions per individual participant) with different intensity-normalization methods (METs, %VO_2max_, and LT). For LT-based normalization 0 = basal VO_2_, 1 = VO_2_ at LT1, 2 = VO_2_ at LT2, 3 = VO_2max._ R² values refer to the marginal pseudo–coefficient of determination for each model. EPOC = excess post exercise oxygen consumption; LnRMSSD = the natural logarithm of root mean square of successive differences iTRIMP = individualized training impulse; Bla = blood lactate; sRPE = session rating of perceived exertion; MET = metabolic equivalents; VO2max = maximum oxygen uptake; LT = lactate threshold.
