# Supplementary material for: Standardizing moderate- and vigorous-intensity exercise doses by physiological strain: an exploratory randomized cross-over study
Source: Eur J Appl Physiol. 2026 Mar 9;126(7):3637–53. doi: 10.1007/s00421-026-06157-1 (PMC13380570; doi:10.1007/s00421-026-06157-1)
Supplement: Supplementary file 3 — Supplementary Material 3 [file 421_2026_6157_MOESM3_ESM.docx]

Standardizing moderate- and vigorous-intensity exercise doses by physiological strain: an exploratory randomized cross-over study

Olli-Pekka Nuuttila^1,2*^, Piia Kaikkonen^,3^, Timi Malinen^2^, Harri Sievänen^1^, Tommi Vasankari^1,4^Heikki Kyröläinen^2^

1 The UKK Institute for Health Promotion Research, Finland

2 Faculty of Sport and Health Sciences, University of Jyväskylä, Finland

3 Tampere Research Center of Sports Medicine, UKK Institute, Finland

4 Faculty of Medicine and Health Technology, Tampere University, Finland

**Corresponding author:**

Olli-Pekka Nuuttila, E-mail: [olli-pekka.nuuttila@ukkinstituutti.fi](mailto:olli-pekka.nuuttila@ukkinstituutti.fi)


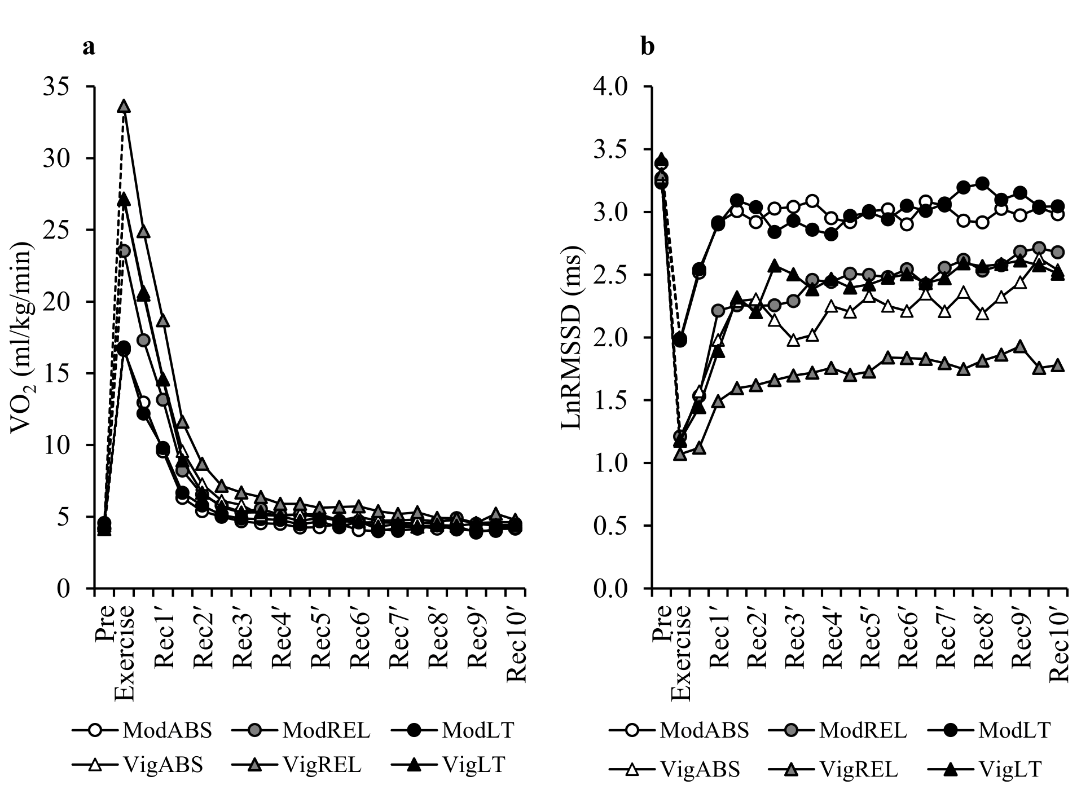


**Electronic supplemental material 3.** Recovery kinetics of oxygen uptake (VO_2_) and natural logarithm of root mean square of successive differences (LnRMSSD) with 30-s averages.
